# Evaluation of a continuous community-based ITN distribution pilot in Lainya County, South Sudan 2012–2013

**DOI:** 10.1186/s12936-017-2020-8

**Published:** 2017-09-11

**Authors:** Albert Kilian, Lisa Woods Schnurr, Tafadzwa Matova, Richmond Ato Selby, Kojo Lokko, Sean Blaufuss, Miatta Zenabu Gbanya, Ruth Allan, Hannah Koenker, Martin Swaka, George Greer, Megan Fotheringham, Lilia Gerberg, Matthew Lynch

**Affiliations:** 1 Malaria Consortium, London, UK; 2 Tropical Health LLP, Montagut, Spain; 3 Malaria Consortium, Juba, South Sudan; 4 Johns Hopkins University Center for Communication Programs, Baltimore, USA; 5 President’s Malaria Initiative, U.S. Agency for International Development, Washington D.C., USA; 6 President’s Malaria Initiative, U.S. Agency for International Development, Dar es Salaam, Tanzania

**Keywords:** Insecticide-treated net, Malaria, South Sudan, Community-based, Insecticide-treated net distribution

## Abstract

**Background:**

Continuous distribution of insecticide-treated nets (ITNs) has now been accepted as one way of sustaining ITN universal coverage. Community-based channels offer an interesting means of delivering ITNs to households to sustain universal ITN coverage. The objective of this study was to provide proof of concept for this channel.

**Methods:**

A 9-month, community-based, distribution pilot was implemented beginning 1 year after a mass campaign in Lainya County, South Sudan from 2012 to 2013. Following social mobilization, community members could request an ITN from a net coupon holder. Eligibility criteria included having lost an ITN, giving birth outside of the health facility, or not having enough ITNs for all household members. After verification, households could exchange the coupon for an ITN at a distribution point. The evaluation was a pre/post design using representative household surveys with two-stage cluster sampling and a sample size of 600 households per survey.

**Results:**

At endline, 78% of respondents were aware of the scheme and 89% of those also received an ITN through community-based distribution. Population access to ITNs nearly doubled, from 38% at baseline to 66% after the pilot. Household ownership of any ITN and enough ITNs (1 for 2 people) also increased significantly, from 66 to 82% and 19 to 46%, respectively. Community-based distribution was the only source of ITNs for 53.4% of households. The proportion of the population using an ITN last night increased from 22.7% at baseline to 53.9% at endline. A logistic regression model indicates that although behaviour change communication was positively associated with an increase in ITN use, access to enough nets was the greatest determinant of use.

**Conclusions:**

ITN access and use improved significantly in the study area during the pilot, coming close to universal coverage targets. This pilot serves as proof of concept for the community-based distribution methodology implemented as a mechanism to sustain ITN universal coverage. Longer periods of implementation should be evaluated to determine whether community-based distribution can successfully maintain ITN coverage beyond the short term, and reach all wealth quintiles equitably.

**Electronic supplementary material:**

The online version of this article (doi:10.1186/s12936-017-2020-8) contains supplementary material, which is available to authorized users.

## Background

With the shift from conventionally treated mosquito nets to long-lasting insecticidal nets (LLIN), which avoid the need for retreatment, insecticide-treated nets (ITNs) have become the most important tool in malaria control in Africa south of the Sahara. Initially distribution strategies were focused on local, often targeted distributions by non-government organizations [1] and some social marketing programmes [2], resulting in only slow improvements in coverage [3]. This changed around 2004 when ITN distributions started to be linked to mass immunization campaigns such as measles [4] or polio [5]. Following the 2007 launch of the new WHO universal coverage strategy for ITNs [6], campaigns changed from being mostly targeted to young children and pregnant women to mass campaigns for the entire population at risk. From 2008 to 2016 more than one billion ITNs have been delivered to African countries and distributed using a variety of approaches to mass campaign organization which in general were successful in rapidly and equitably scaling up ITN coverage if properly managed and implemented [7]. A 2015 study analysing factors contributing to declines in malaria morbidity between 2010 and 2015 found that 68% of the 40% reduction in malaria incidence could be attributed to ITNs [8].

However, it is clear that access to ITNs starts to decrease immediately following a mass campaign, due to births, migration and net loss or damage. Coverage can reduce to levels as low as 40% before the next campaign, potentially resulting in reduction or loss of the community effect [9]. In addition, it is not possible to take existing nets into account during mass campaigns [10], meaning that older but still serviceable nets and those distributed through ante-natal care and immunization services are ignored [6]. These attributes of mass campaigns can result in an oversupply of households with nets [11] and thereby reduce the cost-effectiveness of an ITN distribution strategy that includes repeat mass campaigns.

Comprehensive continuous distribution strategies have been suggested as a mechanism to sustain universal coverage once scale-up is achieved with mass campaigns, and include school and community-based channels in addition to routine distribution via health services and support to commercial market development [12]. However, data are limited about the ability of these strategies to increase or maintain ITN ownership and access. In South Sudan, the malaria control programme and partners distributed approximately 6 million LLINs through mass campaigns between 2008 and 2010 but faced significant challenges due to shortage of staff and weakness of health system infrastructure. Schooling rates in this complex operating environment of South Sudan are low, suggesting that a school-based distribution channel for ITN would not reach enough households to maintain universal coverage. Therefore, the government expressed interest in community-based ITN distribution. The purpose of this pilot study was to test the hypothesis that a demand-driven, community-based ITN distribution scheme would be able to at least sustain the level of coverage achieved through a preceding mass distribution campaign of ITN.

## Methods

### Study site

Lainya County was selected jointly by the South Sudan malaria control programme and implementation partners based on criteria of accessibility, safety and available minimal infrastructure. Lainya County is situated in southern South Sudan in Central Equatorial State with an estimated population in 2011 of 246,000 divided into five *payams* (3rd level administrative unit) and 15 *bomas* (4th level administrative unit). It had at the time one functional hospital, four health centres and 16 health units served by a total of 29 health staff. A mass distribution of ITNs took place in the County in 2010, but the exact number of nets distributed in Lainya County is not available.

### Continuous distribution of LLINs

#### Design

The community-based ITN distribution was based on a ‘push–pull’ system, where ITNs were delivered to a hub near the community (push), here primarily health facilities. Distribution then was driven by the expressed demand from households (pull). Households were informed about the distribution scheme through a social mobilization and communication campaign and encouraged to request new or additional nets if they felt they needed them. Three eligibility criteria were established:

1. An existing ITN that was too damaged and had to be replaced;
2. Less than one ITN for every two people was available in the household;
3. A birth occurred outside a health facility, and/or no ITN was received during ANC services for that pregnancy.

**Fig. 1:**
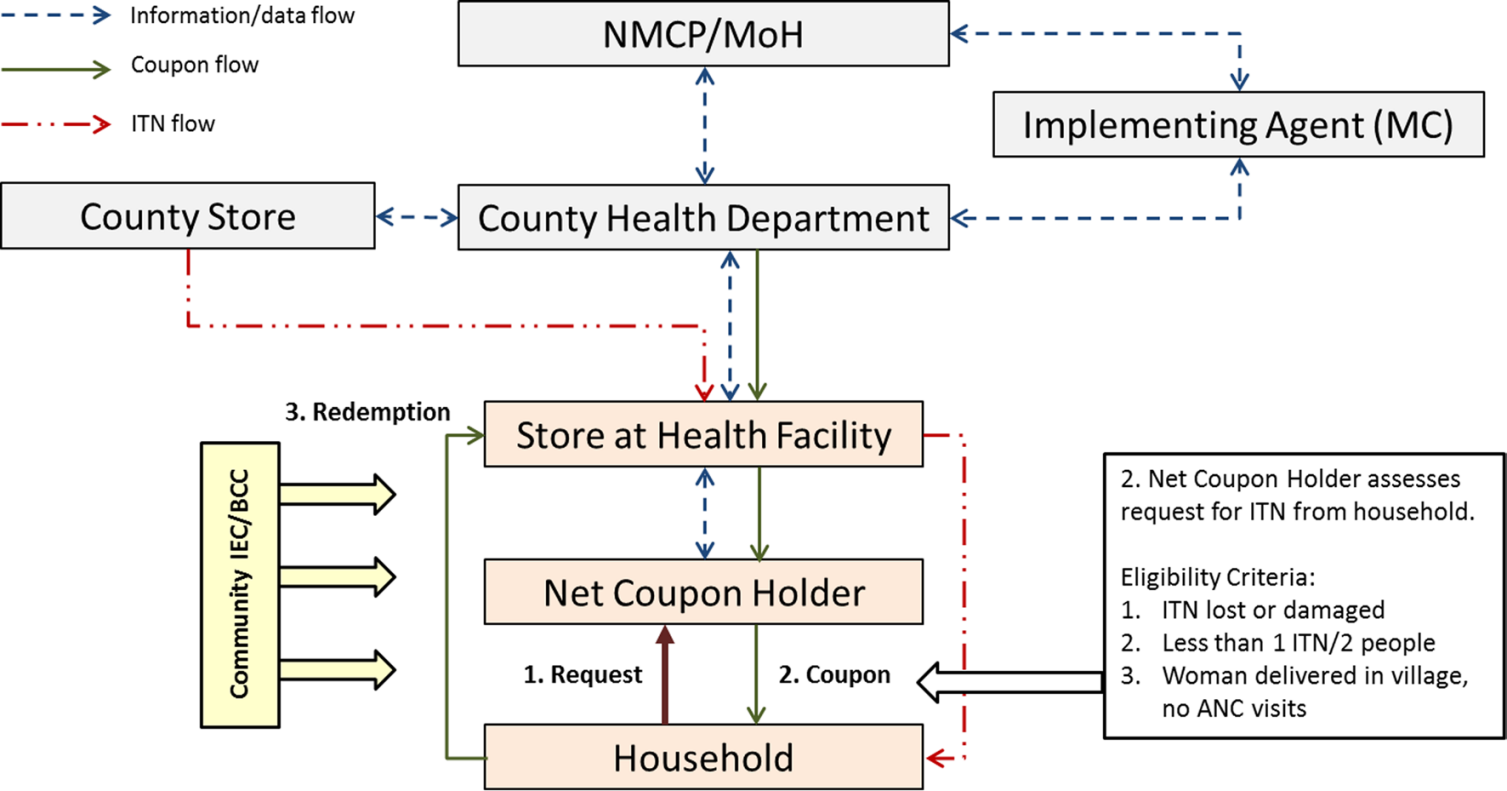
The design and structure of community-based ITN distribution scheme

In order to receive an ITN through the community-based distribution scheme, the household first made a claim to a community agent, called the net coupon holder. The net coupon holder then visited the household and authenticated the claim based on the eligibility criteria. A net coupon holder for every 700 households was trained to support the scheme. After the claim was verified, a coupon was issued to the household. Each coupon was valid for only one ITN but households could be issued multiple coupons. Household members were then able to redeem the coupon for an ITN at a distribution point. Initially, nine health facilities served as distribution points, but five additional remote distribution sites were added to improve distribution point access based on the request of communities to have distribution points closer to them. Since no adequate storage facilities were available at these sites, safe storage boxes were provided. There was then one distribution point for approximately every 2500 households. All distribution sites were supplied by the Lainya County store. Figure 1 illustrates how information, ITNs and coupons flow in the scheme.

### Implementation

The implementation of the scheme was led by the Lainya County health department and the South Sudan malaria control programme. Malaria Consortium provided implementation support and coordination as well as handling funds and initial transport logistics for ITNs. All ITNs were LLINs (PermaNet 2.0 or NetProtect) and were donated by UNICEF and Population Services International (PSI) via Global Fund or procured directly through Malaria Consortium. In addition to the 50 net coupon holders, there were 11 supervisors and 19 social mobilizers identified by the Lainya health department. Each store had a storekeeper and eight stores also had an assistant storekeeper to ensure access at all times. All stores were supervised by the central storekeeper, and monthly supervision and coordination meetings were held between the Lainya County Health Department and the implementing partner.

Activities started in December 2011 with training, social mobilization, and a SBCC (social and behavioural change communication) campaign that included community meetings with involvement of political and faith-based leaders. Coupon issuing started in May 2012 and lasted for 9 months until January 2013, while coupon redemption lasted until mid-March 2013.

### Evaluation

The principle design was a before-after comparison based on household surveys with complementary routine and cost data. There was no external comparison or control group, but a counterfactual was obtained from the endline survey calculating outcomes without the ITNs distributed through the scheme.

### Routine data

Two pieces of data were collected on a monthly basis: the number of ITNs issued at distribution points and the number of net coupons issued by net coupon holders. Net coupon holders and health facilities disseminated information upwards to the County health department, and finally to Malaria Consortium and the Ministry of Health. The data were entered into Excel data sheets. Information on the coupons also included the reason for issuing the coupon based on the three eligibility criteria.

### Household surveys

Representative household surveys were carried out in April 2012 (baseline) and April 2013 (endline) each coinciding with the beginning of the rains, which last from April to October in this area. A two-stage cluster design was used where 30 clusters were selected based on enumeration areas from the 2008 Census and applying probability proportionate to size sampling (population). Within selected clusters, 20 households were sampled by simple random sampling from lists of eligible households compiled by the survey team (Table 1). The sample size of 600 households per survey was calculated to give a precision of ±6%-points for household indicators if the estimate was around 50% assuming a non-response rate of 5% and design effect of 2.0, alpha error of 0.05 and beta-error of 0.2. Sampling of both clusters and households was independently repeated for each round. Surveys were implemented by a local research firm, integrity research and consultancy, using experienced supervisors and interviewers and applying standard measures of quality assurance and supervision. The data collection tool was based on the Malaria Indicator Survey questionnaire with additions to reflect the specific source of nets and the perceptions of the distribution scheme by the population.

**Table 1:** Background characteristics of sampled households (HH)

Category/variable	Baseline		Endline	
	Estimate	95% CI	Estimate	95% CI
Demographics				
Average household size (persons)	5.8	5.5–6.1	5.1	4.9–5.3
HH headed by female	15.3%	12.8–18.3	17.2%	14.0–21.0
Mean age of head of HH (years)	41.5	40.5–42.5	42.0	40.9–43.2
Population <5 years	20.2%	17.6–22.9	15.3%	14.1–16.6
House characteristics				
Thatch or grass roof	96.2%	93.8–97.7	95.5%	92.8–97.4
Mud walls	98.5%	96.0–99.4	97.2%	95.1–98.3
Firewood primary fuel for cooking	93.2%	86.6–96.4	96.3%	92.2–98.3
Average persons/sleeping place	2.0	1.9–2.1	1.5	1.4–1.5
Education of head of HH				
Males non-literate	20.2%	24.8–33.9	30.6%	25.2–36.5
Females non-literate	76.7%	65.9–84.3	58.2%	47.9–67.7
Males secondary or higher	21.9%	18.0–26.5	19.2%	15.6–23.5
Females secondary or higher	2.2%	0.5–8.6	14.3%	8.2–23.7
Water and sanitation				
HH with access to safe water	74.0%	64.8–97.7	75.1%	67.2–82.6
HH with access to any latrine	34.7%	29.1–40.8	55.3%	46.4–63.8
Household assets				
HH owns any radio	45.9%	39.3–52.7	63.6%	57.8–69.1
HH owns any mobile phone	53.7%	47.0–60.3	60.5%	55.2–65.5
HH has any means of transport	51.4%	46.2–56.6	60.3%	54.9–65.5

Data from the paper-questionnaires were double-entered and validated using CSPRO software and then transferred for analysis to the Stata software package (version 11). Chi squared test was used for dichotomous or grouped variable comparisons and Student’s t test or non-parametric Kruskal–Wallis test for continuous variables, always adjusting for the cluster-design of the survey. In order to account for potential confounders in the analysis of key outcomes, multivariable regression models were used, logistic regression for dichotomous outcomes and linear regression for continuous variables. Models were constructed using backwards elimination and Wald tests for significant parameters.

Primary outcome measures were those of net and ITN ownership as recommended by the Roll-Back Malaria Monitoring and Evaluation Reference Group (RBM-MERG) namely the ‘proportion of households with any ITN’, the ‘proportion of households with at least one ITN for every two people in the household’ (considered to be enough so that every member could use an ITN) and the ‘proportion of the population with access to an ITN within the household’ assuming each ITN is used on average by two people [13]. Counterfactual ownership coverage was calculated by only using nets identified as from the campaign or routine distribution, respectively. In the same way, the 2011 post-campaign coverage was estimated from the number of campaign nets received from the campaign reported at the baseline survey.

To evaluate equity of net ownership indicators, a wealth index was created applying principal component analysis (PCA) and using household amenities, livestock, assets, and other characteristics relevant to household socio-economic status in South Sudan as input variables. The first component was used as wealth index and households grouped into wealth quintiles. Concentration indices were calculated to quantify equity. The concentration index utilizes data from all wealth quintiles and expresses perfect equity as 0, maximum ‘pro-poor’ inequity as −1 and maximum ‘pro-rich’ inequity as +1. Standard errors and confidence intervals for the concentration indices were calculated using the formula suggested by Kakwani et al. [14].

Community-level ITN coverage was estimated for each survey cluster applying the Lot-Quality-Assurance-Sampling-based methodology (LQAS) suggested by Biedron et al. [15]. In short, each cluster was considered a lot and a pass-fail assessment was made for different ITN coverage targets based on LQAS decision rules based on a 20%-point margin between target and minimally acceptable result (90/70, 80/60% etc.).

Data on programme costs were collected and made available by Malaria Consortium’s finance department. Costs were categorized as either direct costs, indirect costs or evaluation costs. These were further divided into sub-categories following WHO recommendations and previous costing work by Kolaczinski et al. [16]. Costs were initially reported in GBP, and were converted to USD using an average exchange rate for the period of USD 1.60 per GBP. No cost from the County or national level from the Government of The Republic of South Sudan were available for inclusion so that costing only reflects funding by the donors (donor perspective). In addition, only financial and not economic cost estimated was used.

## Results

### Coupons and ITNs issued

**Fig. 2:**
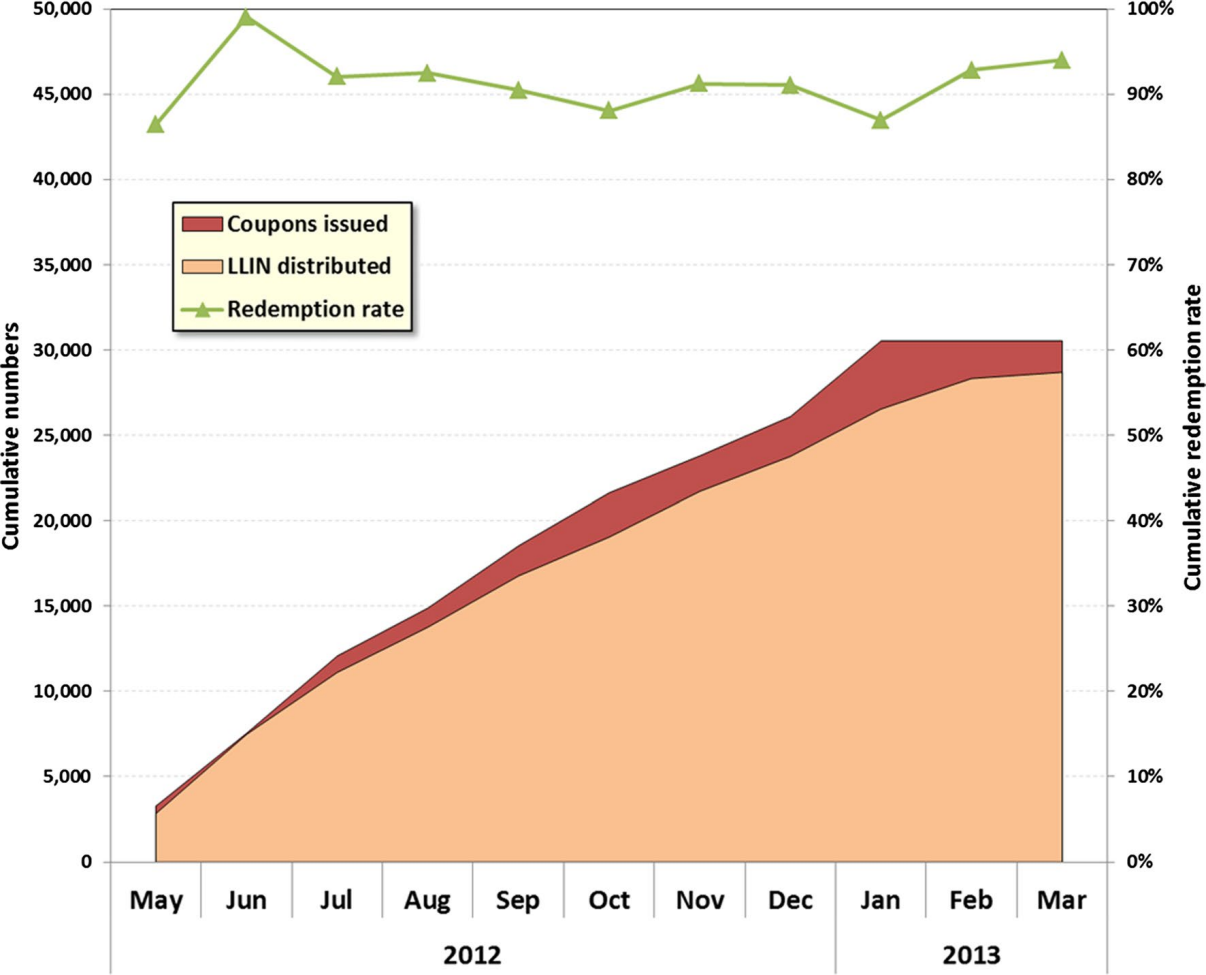
Number of coupons and LLINs issued and coupon redemption rate

Over the 11-month pilot a total of 30,530 coupons were issued to beneficiaries and 28,696 ITNs were distributed, resulting in an overall redemption rate of 94.0%. As shown in Fig. 2, output was quite homogeneous over time with on average 3188 ITNs issued per month. Redemption rate varied between 99.1% in the second month and 86.9% in January 2013 when the issuing of coupons ended. For 81.5% of coupons a reason for issuing was stated: 57.1% were given because there were not enough ITNs in the household; 31.8% to replace damaged nets; and, 11.2% to women who had given birth but had not received an ITN. Overall, 18.5% of coupons did not have a reason recorded and neither this rate nor the distribution between the recorded reasons varied significantly during the course of the pilot.

### Evaluation surveys

Out of the targeted sample size of 600 households the baseline survey achieved 599 and the endline 597. The corresponding numbers of household members registered in these households were 3567 and 3095, respectively. The setting is a very rural environment where almost all houses have a thatched roof and mud walls, the predominant fuel for cooking is firewood, and the educational level of the mainly male heads of household is relatively low. About three-quarters of the households had access to safe drinking water, mainly from boreholes, and one-third at baseline and half at endline had access to any form of latrine. Both access to safe water and latrines was strongly associated with increased wealth (p < 0.001). Household assets such as radios, mobile phones or means of transport also showed a strong poor-rich gradient (p < 0.001) but also some improvement between the two surveys 12 months apart. The reason for this increase in assets is not clear but did not change the poor-rich gradient from baseline to endline survey. There were also some demographic differences between the samples with a slightly lower average household size at endline (p < 0.05), lower proportion of children under five in the population (p < 0.05) and subsequently a lower person-to-sleeping place ratio (p < 0.05). From the data it cannot be concluded with certainty whether these were based on changes in population composition (e.g., due to migration) or slightly different composition of the sample by chance.

### Access to ITNs via the community-based distribution

At endline 78.3% of households were aware of the continuous distribution scheme. Community members had heard about it through social mobilizers (77.0%), net coupon holders (71.3%), distribution supervisors (28.9%), clergymen (21.7%), and/or health workers (18.5%). *Boma* chiefs, village health committee members, neighbours, and other officials were reported as sources of information by less than 10% of households. Most households aware of the scheme also requested an ITN coupon during the 9 months of operation (95.5%); most of these (95.6%) received at least one coupon, almost all of those (99.1%) went to redeem the coupon, and 98.1% of those also received an ITN. As shown in Table 2, the overall community effectiveness of households receiving any ITN through the scheme was 69.9, and 88.7% of those aware of the campaign ended up receiving at least one ITN through the scheme. Stratifying by wealth quintile, there was a certain level of pro-rich inequity (p = 0.003), which had its basis in a lower number of the poorest wealth quintiles hearing about the scheme and then increasing at each step slightly. But as seen in Table 2 from the concentration indices this inequity was moderate and receiving an ITN if aware of the scheme was quite equitable (concentration index 0.03) even though the trend by wealth quintile was still statistically significant.

**Table 2:** Effectiveness and equity of community-based distribution scheme components

Background characteristic	Heard about coupon	Requested coupon	Received coupon	Went to redeem coupon	Received ITN	Received ITN if heard about coupon
Endline						
Estimate (%)	78.3	75.2	71.9	71.2	69.9	88.7
95% CI	72.6–83.8	68.3–81.1	64.1–78.5	63.4–77.9	61.6–77.0	82.4–93.0
Wealth index						
Lowest (%)	67.2	60.5	56.3	55.5	53.8	80.0
Second (%)	79.8	74.8	72.3	71.4	70.6	88.4
Third (%)	75.0	72.5	68.3	66.7	64.2	85.6
Fourth (%)	83.2	79.8	77.3	77.3	77.3	92.9
Highest (%)	88.3	88.3	85.0	85.0	83.3	94.3
p value Chi squared for trend	0.003	<0.001	<0.001	<0.001	<0.001	0.003
Concentration index						
Estimate	0.046	0.065	0.070	0.073	0.075	0.029
95% CI	0.023–0.070	0.038–0.091	0.049–0.098	0.044–0.102	0.046–0.105	0.010–0.048

Only 20 of the 597 households surveyed at endline reported not receiving a coupon despite attempting to acquire an ITN. Of those, thirteen were unable to find a net coupon holder, four households were ineligible based on the established criteria, and four were uncertain why they had not been issued a coupon. Eight households unsuccessfully attempted to obtain nets from distribution points. Four of the eight came when the person responsible for distribution was unavailable, three came during stock outs, and one arrived after the closure of a health facility.

### ITN ownership and use—before and after

At baseline, 773 mosquito nets were registered in surveyed households of which 91.5% could be identified as LLINs based on brand, three (0.4%) were reported as having been dipped with an insecticide within the last 12 months and 8.1% were untreated nets. At endline there were 1251 nets registered with 99.0% identified as LLINs and 1.0% untreated. The changes in ownership and use indicators are shown in Table 3 and demonstrate significant improvements in all areas. As one would expect, the smallest increase of 1.2-fold (from 66.3 to 81.7%) was seen in the proportion of households with any ITN, while the population with access to an ITN within the household almost doubled with a 1.8-fold increase (37.9 to 66.2%). The proportion of households with enough ITNs for all members (at least one ITN for every two people) increased 2.5-fold (from 18.5 to 45.6%). This was accompanied by significant reduction in households with some, but clearly not enough ITNs (<1 ITN for every three people) from 52.9% at baseline to 23.2% at endline. In contrast, the proportion of households with more than enough ITNs remained low (4.5 and 8.8%, respectively).

**Table 3:** Net and ITN ownership and use before and after the community-based distribution

Category/indicator	Baseline		Endline	
	Estimate	95% CI	Estimate	95% CI
Household ownership				
Proportion with any net	74.3%	67.6–80.0	82.4%	77.4–86.5
Proportion with any ITN	66.3%	60.1–71.9	81.7%	76.8–85.8
Proportion with at least 1 ITN/2 people	18.5%	15.2–22.5	45.6%	39.2–52.0
Mean number of ITNs if any owned	1.79	1.65–1.92	2.54	2.39–2.68
Supply with ITN if any owned				
<1 ITN/3 people	52.9%	47.3–58.4	23.2%	17.7–29.6
1 ITN/3 people	19.1%	14.8–24.4	21.1%	17.4–25.3
1 ITN/2 people	23.4%	18.9–28.6	46.9%	41.1–52.9
1 ITN/person or more	4.5%	2.8–7.3	8.8%	6.2–12.4
Population (de-facto) access				
Proportion with access to an ITN	37.9%	33.3–42.7	66.2%	61.2–70.9
Population use of ITNs				
Used ITN last night	22.7%	18.0–28.3	53.9%	49.6–58.2
Used ITN if access	60.1%	55.3–64.9	81.5%	76.4–86.1

The proportion of the population using an ITN last night increased from 22.7% at baseline to 53.9% at endline. The proportion of household members using an ITN if they had access within the household increased 1.4-fold reaching 81.5% at endline (Table 3). In addition, there was a clear increasing prioritization of ITN use from baseline to endline for children under five and women in reproductive age. Dividing the population into three groups (under-fives, women age 15–49 years, heads of household) and comparing these against the rest of household members, a logistic regression analysis adjusted for wealth and whether a household owned enough ITNs for all members showed an adjusted odds ratio (OR) for ITN use the previous night for children under five of 2.3 at baseline (95% CI 1.6–3.4) increasing to 5.1 (3.7–7.1) at endline. The increase for women in reproductive age was not quite as strong with adjusted OR 2.2 (1.7–3.0) at baseline and 2.9 (2.–3.6) at endline. Heads of households were also more likely to use an ITN with the adjusted OR increasing from 2.6 (2.1–3.3) at baseline to 3.7 (2.9–4.8) at endline. Other factors that had a positive association with ITN use at endline were discussing net use in the household (adjusted OR 6.1, 4.7–13.7) and the expressed intention to use the nets every day (adjusted OR 3.1, 1.2–7.8). The latter were linked to behavioural change communication (BCC) activities.

### Behaviour change communication

Social mobilization began three months prior to the launch of the pilot, and net coupon holders and social mobilizers delivered ITN messaging throughout the pilot. At baseline, 44.2% of all households reported receiving information on ITNs or ITN use, with health workers (33%) and radio (28%) as the two most common sources of information. Over the course of the pilot, social mobilizers (65%) and net coupon holders (58%) became the primary sources of ITN information for all households. Overall, 58.1% of households had received ITN information by the end of the pilot, a statistically significant increase (p < 0.05). Among households who had actively participated in the community distribution scheme, 79% of household who obtained at least one net coupon through this channel were exposed to ITN messaging.

Households exposed to ITN messaging were significantly more likely to have discussed net use (98 vs 56%, p < 0.0001) and expressed their intention to use an ITN regularly (95 vs 48%, p < 0.0001). As has been presented above, these two variables were also directly linked to actual use suggesting that the BCC activities in combination with ITN availability contributed to the increased ITN use.

### Perceptions of community-based distribution scheme

Among households who were aware of the distribution scheme, 63.6% felt that the distance to the net coupon holder was either acceptable or very close. The remaining 36.4% believed the nearest net coupon holder was too far. Distribution sites were perceived to be slightly less accessible, with only 47.8% of participants deeming sites as very close or an acceptable distance. Of the five *payams*, central Lainya *payam* households generally felt that both net coupon holders and distribution sites were more accessible than the four peripheral *payams*. Perception of distance was also affected by whether or not a household participated in the community distribution scheme, with 63% of those who were aware of the campaign but elected not to pursue a coupon feeling that net coupon holders were not close enough, while only 35% of those who requested and received a coupon felt the distance was too far (p = 0.04).

The communities and households largely felt that the criteria for eligibility were fair, with 88.0% replying very fair (63.2%) or fair (24.8%). Similar to perceived distance to a net coupon holder, this was also skewed by participation in the distribution scheme. Only 61% of households who did not receive a coupon felt the criteria were fair, compared to 91% among those who did receive a coupon (p < 0.005).

### Equity

**Fig. 3:**
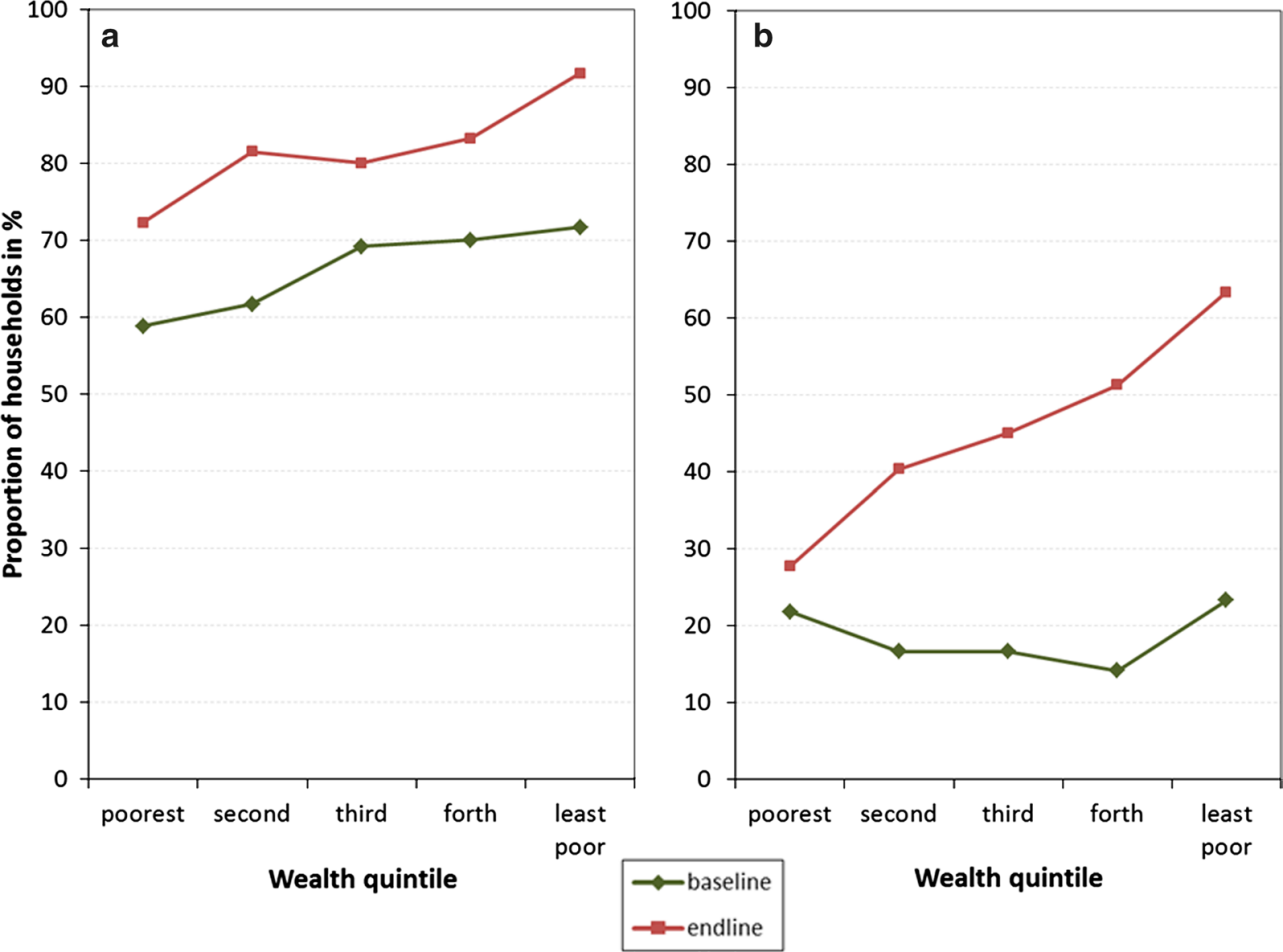
Household ownership of any nets (**a**) and enough nets (**b**) by wealth quintile

As presented in Table 2, households in the poorer wealth quintiles were less likely to request a new ITN through the community-based distribution. The poorest quintile were as likely as wealthier quintiles to receive an ITN if they received a coupon, but much less likely to know about community distribution, or request a coupon if they did know about the scheme than the wealthier quintiles. A closer look at equity of ITN ownership is presented in Fig. 3, showing that the gradient between wealth quintiles for coverage with at least one ITN remained nearly unchanged between baseline and endline survey, i.e., the curve only shifted upward. However, equity for enough ITNs (at least one ITN for two people) showed a significantly more pro-rich distribution. This is also reflected in the concentration indices which were identical, 0.041 and 0.040, for households owning any ITN at baseline and endline, respectively. The concentration indices increased from 0.002 to 0.144 (p < 0.05) for households with enough ITNs.

### Community coverage

**Fig. 4:**
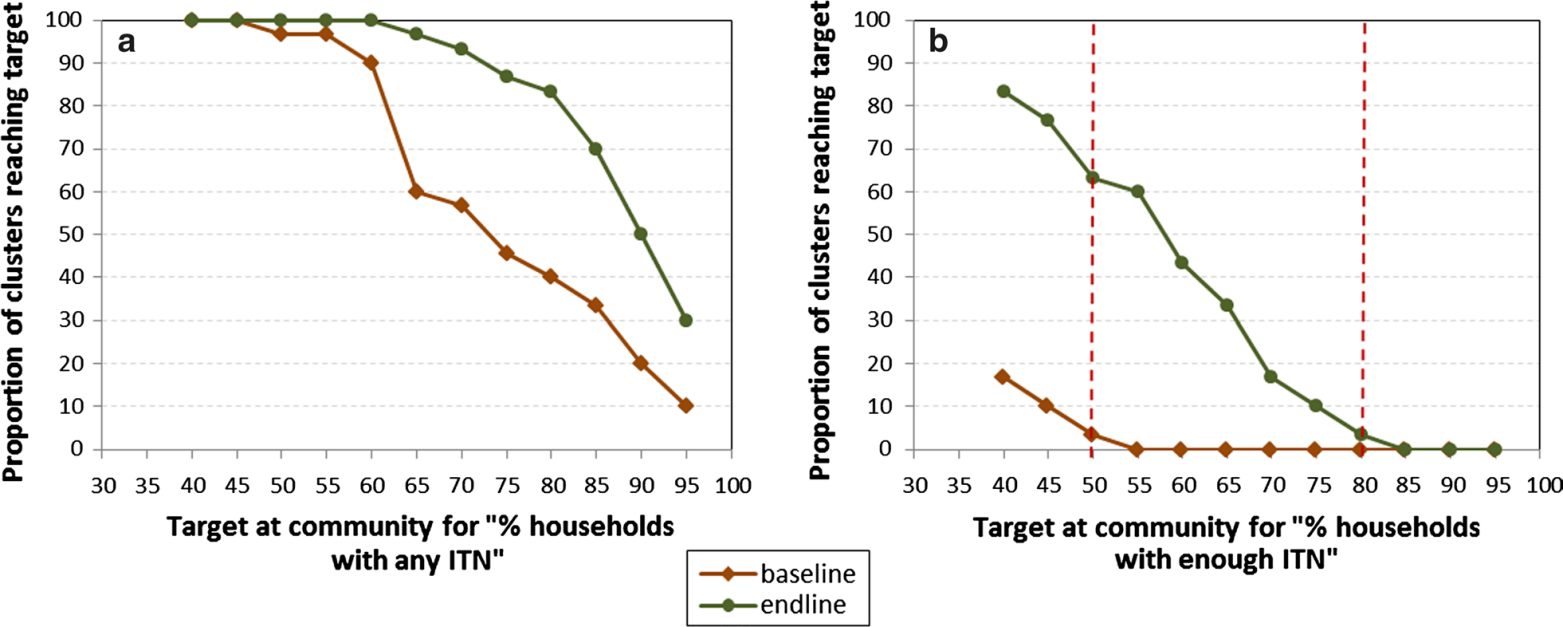
Community level coverage estimates for households with any ITN (**a**) and enough ITN (**b**)

Results from the LQAS approach to assess community-level coverage are presented in Fig. 4, showing that community-based distribution substantially raised community coverage for household ownership of any ITN, and households with enough ITNs. At baseline, nearly all (>95%) communities achieved a level of 50% of households with at least one ITN, but only 40% of communities attained the national target of 80% household ITN ownership. Almost none (3%) of the communities surveyed at baseline met the target of having 50% of households with enough ITNs. At endline, about 85% of communities were found to have over 80% household ownership of at least one ITN, and more than 60% of communities had half of households owning enough ITNs. As Fig. 4 shows, there were noticeable gains at each five percentage point marker up to 80% for households with enough ITNs.

### Other sources of nets and ownership trends over time

At endline, 69.9% of households had received an ITN through the community-based distribution scheme, and 95.9% of those still owned these nets. Other distribution channels that households received ITNs from included routine ante-natal care services (ANC) (15.4%), the previous mass distribution campaign (12.4%), and the retail market (10.1%). Utilization of the community-based distribution scheme varied based on whether a household did or did not receive ITNs through the previous mass campaign, with 70% of households that did not receive any ITNs through the mass campaign receiving ITNs through community-based distribution, compared to 50% of households that did receive nets through the prior mass campaign. Households with women attending ANC at a health facility were less likely (56%) to receive an ITN through community distribution than households not attending ANC. Following the mass campaign, the community-based distribution served as the only source of new ITNs for more than half of households (53.4%). Nets from multiple channels were obtained by only 15% of households and the most common combination was community scheme plus ANC (8.4%) followed by community scheme plus nets bought from private sector (5.7%). Only 6% received new ITNs only from ANC.

**Fig. 5:**
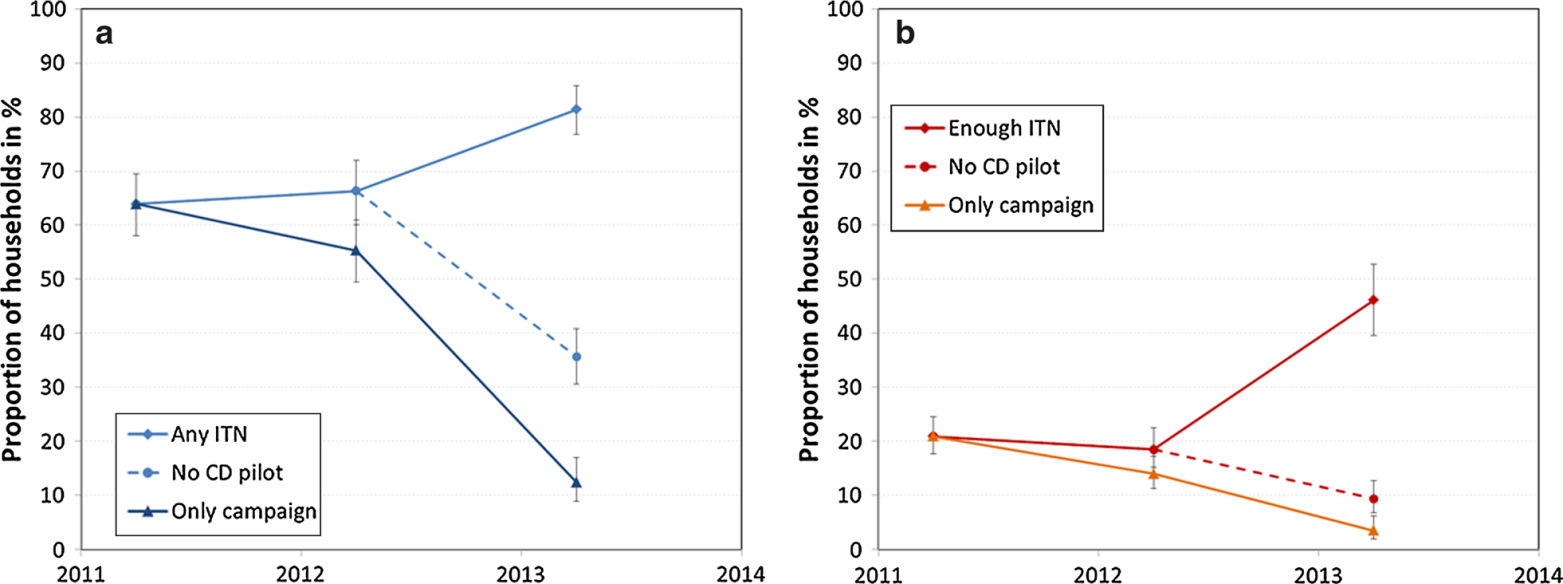
ITN ownership with and without community-based distribution for any ITN (**a**) and enough ITN (**b**)

The trend in ITN ownership coverage from the campaign in 2011 up to the endline survey in 2013 is shown in Fig. 5, giving the overall result as well as the counterfactual of what coverage would be observed without the contributions of the CD pilot, and only considering ITNs remaining from the campaign and private sector. The 2011 post campaign results are estimated from the baseline survey based on campaign nets received reported by the households. Results show that the proportion of households with any ITN increased moderately from 64% at the time of the mass campaign to 66% at the start of the CD-pilot and then increased sharply to 81% at the endline survey. In contrast, the proportion of households with at least one ITN for every two people slightly decreased after the campaign from 21 to 19% but then sharply increased to 46% at endline. Excluding community-based distribution would have resulted in coverage of 36% with any ITN and only 9% with at least one ITN for every two people in 2013. There also was a clear difference between only campaign nets and coverage without the community-based distributions showing that nets from ANC, private sector and ‘other sources’, i.e., friends, family and civil society, contributed to post-campaign coverage but not enough to prevent decline of coverage rates.

### Cost

The financial cost of the community-based distribution scheme in Lainya County was composed of direct, indirect and household survey costs. Total cost for all three categories was USD667,299 and USD551,318 after removing the cost of surveys resulting in cost per ITN distributed of USD19.21 and USD16.52 when the procurement of the ITN was excluded. Indirect cost for staff employed by the implementer to support the scheme and their travel and management cost was high and made up 49% of the total. Direct cost alone was USD9.79 per net delivered and USD7.02 without ITN procurement. Of the direct cost the largest proportion (58.7%) went to operations with transport (vehicle hire and fuel) being the largest component with 27.6% of direct cost followed by training, mobilization and BCC (12.1%), net storage (9.3%) and supervision (7.8%). Incentives for the storekeepers, including airtime for communication, comprised 9.4% of direct cost (details of cost categories are shown in Additional file 1).

Plotting cumulative cost per net delivered against the months of programme operation showed a very high initial cost based on the high spending during roll-out of USD31.30 (without ITN procurement) in the first month which then rapidly declined in the next 3 months to USD10.59 and then slowly further declined to reach a steady state around USD6.55 after 9 months of implementation.

## Discussion

Community-based distribution of ITNs in Lainya County over a period of 9 months in conjunction with additional ITN distribution through ante-natal care services (ANC) and purchases from the retail market by some households significantly increased population access to ITNs from 38% one year after a mass distribution campaign to 66% at the end of the pilot. Household ownership of any ITN at endline was 82% and 46% of households owned enough ITNs for all household members (one ITN for every two people). In spite of the increases the target for universal coverage of 80% population ITN access was not quite achieved. This was in part due to the lower than expected coverage remaining from the mass campaign of 2010. Awareness and acceptability of the scheme were high. In addition, the endline survey results showed a high level of community coverage where 85% of communities (clusters) had at least 80% of households with any ITN and 60% had at least half of households with enough ITNs for all household members. Although the exact minimal and optimal coverage levels for the ITN mass effect are not precisely defined, available evidence suggests [17, 18] that the observed community coverage level is sufficient for a significant impact on transmission reduction and protection of people without access to an ITN, assuming there is no pyrethroid resistance among the dominant vectors.

A number of descriptions of continuous, community-based net or ITN distributions can be found in the literature to which the Lainya pilot can be compared. However, most of these were undertaken before universal coverage became the objective of malaria prevention with ITNs. These distributions were either part of implementation research, smaller scale projects or social marketing approaches. Community health workers or community leaders have been used to selling subsidized nets in Afghan refugee camps in Pakistan [19], in several communities in Latin America [20], and in social marketing projects in Kilombero, Tanzania [2] and Sofala and Manica Provinces in Mozambique [21]. Community-based organizations, such as committees or cooperatives, have been used for the same purpose in four areas in Kenya [22], and Mexico and Colombia [23]. All these examples show that a demand-driven ITN distribution scheme is feasible and acceptable to the communities, but using these approaches alone and without building on a previous mass campaign, ITN ownership only increases gradually [3]. Only one paper describes the outcome of a community-based ITN distribution that had the objective to reach universal ITN coverage. In a health district in Western Niger community health committees were supported to sell subsidized ITNs (USD1.20 to the consumer) to all interested households resulting in ITN ownership of at least one ITN of 83% compared to 61% in a control area [24]. Given that ITNs were sold and not given free as in Lainya County, this success is very similar to what was reported in this study even though not all ownership indicators are reported and no baseline was undertaken.

The present pilot provides proof-of-principle that a community-based ITN distribution scheme can reach sufficient number of households to sustain and even increase ITN coverage thereby confirming the study hypothesis. This is demonstrated by several findings. First, with 96% of households who were aware of the scheme requesting additional ITNs at least once, households showed that they not only were aware of the need for more nets, but also were ready to take the initiative to get them. Second, after the addition of distribution points in remote areas, the density and functionality of access points was sufficiently high for people to obtain ITNs. Less than 1% of households received a coupon but did not go to a redemption point, and only another 1% did not get a net because of stock-out or other reasons, resulting in a 94% redemption rate. This suggests that acceptability in the community was high enough for this system to continue at a similar level provided demand was met with supply. The lower awareness of the scheme among households in the lowest wealth quintile, however, is more problematic. This resulted in a pro-rich gradient especially for the indicator for ‘enough’ ITNs for all household members (concentration index 0.14). Although the inequity was moderate compared to other observations on ITNs or immunization coverage from African countries [25], it was significant enough to raise concern and in contrast to the high level of equity generally found following mass campaigns [3, 7]. Future interventions should establish additional social mobilization targeted especially at this group to ensure they are empowered to request sufficient numbers of ITNs to protect their families keeping in mind, however, that this is likely to further increase cost. Third, there were no attempts observed to trick the system or fraudulently obtain nets and 88% of households thought the system to be fair or very fair.

Lainya County is a challenging, rural operating environment with limited resources and a poorly functioning health system. Accordingly, significant efforts were needed by the implementation partner to initially set the scheme up and provide the necessary supervision and support to run it, resulting in an overall direct and indirect financial cost from the donor perspective of USD16.50 per net delivered without ITN procurement. At a steady state after 9 months, the cost was around USD6.50 per net delivered which is still significantly higher than the USD1.00–1.50 found for mass campaigns [10, 16, 26]. However, these cost estimates may not be comparable for a number of reasons. First, cost estimation methodologies have been found to lack standardization [27], which is especially true for the levels of contributions from other sources than the donor, such as staff time of government employees or use of government vehicles. In the case of the pilot described here these contributions were minimal, thereby increasing the contribution by the donor. Second, although higher than the cost of an average mass campaign, cost of the pilot were not far from those found for a variety of non-campaign distribution approaches such as health facility-based schemes and social marketing where the annual economic costs for an assumed 3 year life span of the nets ranged from USD3.50 to 7.75 [28]. In addition, it can be expected that in the difficult environment of South Sudan cost for a mass campaign would also exceed the figures quoted above. Unfortunately, such cost estimate is not available. Third, the primary objective of the pilot was to provide evidence that a community-based approach can work in a difficult environment. Supervision was intense: cars were hired from the private sector, and incentives were given to storekeepers to ensure they were motivated to participate in an activity that was not part of their routine tasks at the time. Finding the optimal point between increased efficiency through operational cost savings and the danger of decreased cost-effectiveness through diminished impact is always a challenge and in general this process occurs during routine implementation and not at the pilot stage. While it is likely that in such routine implementation donor cost per net delivered would further decrease, it is impossible to say by how much. Finally, one must keep in mind that the cost presented here is only the financial cost of delivery from the donor perspective and do not take into account programme effects and benefits over the lifetime of the ITN. A recent analysis of system efficiencies of ITN distributions in Africa suggests that up to 21% of ITNs may be over-allocated by the current practice of mainly repeat campaigns with some distributions through routine health services [11]. It is, therefore, possible that a continuous distribution through a community scheme that builds on a push–pull mechanism may reduce such inefficiencies. However, cost–benefit analysis in a routine implementation setting would be needed to provide such evidence.

There were two key limitations of this study. As mentioned previously, during the implementation of this pilot special efforts were put into regular supervision and monitoring. This prevents generalizing the positive results to more routine settings or implementation within the general health system where high motivation of staff and additional resources may not be available. Secondly, the relatively short length of the pilot (9 months) precludes drawing any conclusions about the longer-term ability of the scheme to maintain ITN ownership and access.

## Conclusion

The Lainya County pilot provides evidence that a demand-driven community-based distribution can not only sustain ITN coverage levels obtained through a mass campaign but also increase population ITN access across communities and improve the number of households with enough ITNs for all household members. Longer periods of implementation should be evaluated to determine whether community-based distribution can successfully maintain ITN coverage beyond the short term, and reach all wealth quintiles equitably.

## Additional file

**Additional file 1.** Detailed cost of pilot implementation.
