# Supplementary material for: Evaluation of a continuous community-based ITN distribution pilot in Lainya County, South Sudan 2012–2013
Source: Malar J. 2017 Sep 11;16:363. doi: 10.1186/s12936-017-2020-8 (PMC5594500; doi:10.1186/s12936-017-2020-8)
Supplement: Supplementary file 1 — Additional file 1. Detailed cost of pilot implementation. [file 12936_2017_2020_MOESM1_ESM.docx]

**Additional File 1**: Detailed cost

Table: Direct and indirect cost of net delivery from donor perspective

| **Category** | **Item** | **USD** | | **% of direct/**  **indirect** | **% of total** |
| --- | --- | --- | --- | --- | --- |
|  |  | **Cost** | **Cost/net** |  |  |
| **Direct cost** |  |  |  |  |  |
| Commodity | Coupon booklets | 7,217.01 | 0.25 | 2.6% | 1.3% |
|  | Printer | 590.02 | 0.02 | 0.2% | 0.1% |
|  | Metal boxes and locks | 647.38 | 0.02 | 0.2% | 0.1% |
|  | Registry books | 3,767.12 | 0.13 | 1.3% | 0.7% |
|  | ***Sub-Total*** | ***12,221.52*** | ***0.43*** | ***4.3%*** | ***2.2%*** |
|  | LLIN procurement | 77,387.55 | 2.70 | 27.5% | 14.0% |
|  | ***Sub-Total*** | ***89,609.07*** | ***3.12*** | ***31.9%*** | ***16.3%*** |
| Operation | Design and planning | 3,670.02 | 0.13 | 1.3% | 0.7% |
|  | LLIN storage and transport | 26,127.78 | 0.91 | 9.3% | 4.7% |
|  | Training, mobilization & BCC | 34,104.86 | 1.19 | 12.1% | 6.2% |
|  | Supervision | 21,788.59 | 0.76 | 7.8% | 4.0% |
|  | Stationary & telephone etc. | 1,667.44 | 0.06 | 0.6% | 0.3% |
|  | ***Sub-Total*** | ***87,358.69*** | ***3.04*** | ***31.1%*** | ***15.8%*** |
|  | Transport (fuel, car hire etc.) | 77,511.22 | 2.70 | 27.6% | 14.1% |
|  | ***Sub-Total*** | ***164,869.90*** | ***5.75*** | ***58.7%*** | ***29.9%*** |
| NCH & storekeepers | Incentives | 21,508.80 | 0.75 | 7.7% | 3.9% |
|  | Support (phone airtime, stationary etc.) | 4,986.21 | 0.17 | 1.8% | 0.9% |
|  | ***Sub-Total*** | ***26,495.01*** | ***0.92*** | ***9.4%*** | ***4.8%*** |
|  | **Total w/o LLIN purchase** | **203,586.43** | **7.09** | **72.5%** | **36.9%** |
|  | **Total Direct** | **280,973.98** | **9.79** | **100.0%** | **51.0%** |
| **Indirect cost** |  |  |  |  |  |
|  | Staff | 112,816.37 | 3.93 | 41.7% | 20.5% |
|  | Benefits and per diem | 79,389.22 | 2.77 | 29.4% | 14.4% |
|  | Office cost | 36,750.18 | 1.28 | 13.6% | 6.7% |
|  | Travel | 27,846.66 | 0.97 | 10.3% | 5.1% |
|  | Management | 13,541.87 | 0.47 | 5.0% | 2.5% |
|  | **Total Indirect** | **270,344.29** | **9.42** | **100.0%** | **49.0%** |
|  | **Total w/o LLIN procurement** | **473,930.72** | **16.52** | **175.3%** | **86.0%** |
| **Total** |  | **551,318.27** | **19.21** |  | **100.0%** |
